# Sensors Without Boundaries: Innovating Health Monitoring

**DOI:** 10.3390/s25175459

**Published:** 2025-09-03

**Authors:** Sergey Shleev, Magnus Falk

**Affiliations:** 1 Department of Biomedical Science, Malmö University, 20506 Malmö, Sweden; 2 Biofilms Research Center for Biointerfaces, Malmö University, 20506 Malmö, Sweden; 3 Citizen Health Research Center, Malmö University, 20506 Malmö, Sweden

In recent years, we have witnessed a convergence of disciplines in physiological monitoring. Advances in microelectronics, materials science, data analytics, and biotechnology are coalescing to produce (bio)sensors that seamlessly track the body’s signals in real time. These developments are transforming healthcare and human performance: wearable devices now monitor vital signs continuously, and intelligent algorithms sift through sensor data to detect health issues early. The result is a more personalized, proactive approach to well-being, where continuous monitoring and feedback can improve outcomes and quality of life [1]. These advances highlight the inherently interdisciplinary and even transdisciplinary nature of modern biosensing, underlining the importance of broad collaboration. With this Special Issue, we extend an invitation to researchers and practitioners to engage with its diverse contributions and continue innovating across boundaries to push the frontiers of (bio)sensing for global health.

## 1. Embracing Diversity and Ambition in Biosensing

The contributions in this Special Issue exemplify the diversity and ambition of the field. Spanning electrochemical biosensors for biochemical detection, optical and photonic systems for physiological monitoring, and wearable devices seamlessly integrated into daily life, the papers collectively highlight how sensor technology is advancing on multiple fronts. The collection also showcases the power of AI-driven systems that interpret complex biosignals and integrated platforms that combine multiple sensing modalities. The applications are equally broad-ranging, from glucose monitoring and diagnostic testing to neural research, social neuroscience, and operational safety for divers. This further underscores that modern sensor innovation is inherently interdisciplinary. At Malmö University—where interdisciplinary [2], and even transdisciplinary [3], health technology research thrives—we are inspired by the way global teams of engineers, clinicians, neuroscientists, and data scientists are collectively pushing the boundaries of what (bio)sensors can achieve.

## 2. Broadening the Definition of (Bio)Sensor

In this Special Issue, we use the term “(bio)sensor” in an inclusive sense. Traditionally, a biosensor is defined by the incorporation of a biological recognition element (e.g., an enzyme or antibody) for analyte detection. Here, however, “(bio)sensor” refers broadly to any sensor aimed at monitoring physiological signals, regardless of the nature of its recognition mechanism (biological or not). This broadened definition reflects the modern landscape of physiological monitoring, where innovations range from biochemical detectors to physical transducers, all serving the common goal of capturing health data.

## 3. Towards Personalized and Preventive Healthcare

The synergy of these technologies heralds a new era of personalized and preventive healthcare. Continuous, real-time data from wearable (bio)sensors—often enhanced by artificial intelligence—enable the early detection of physiological changes and disease indicators that were once easily missed [1]. Wearable sensors can now measure both physical states and biochemical markers in the body, providing a holistic view of individual health. AI-driven analysis distills these data streams into actionable insights, improving clinical decision-making and patient care [4]. Such approaches hold great promise for reducing healthcare costs and improving quality of life globally. The integration of multiple sensor types into cohesive platforms means that no signal is considered in isolation, paving the way for a more comprehensive understanding of human physiology.

## 4. Diverse Disciplinary Contributions

In this Special Issue, papers from a diverse range of research domains are presented. Contributions span human physiology (focusing on autonomic and cognitive functions, e.g., Freiberger et al., where heart rate variability features in divers were linked to impaired cognitive performance under narcotic gas exposure, suggesting their potential as early warning markers), neurophysiology (including aging-related physiology, geriatric cardiovascular function, and cognitive neuroscience, e.g., Xue et al., where continuous monitoring and functional data analysis revealed age-, BMI-, and orthostatic hypotension-related differences in cardiovascular and cerebral oxygenation responses during orthostatic challenge), sensor development (covering neural signal amplifiers, wearable biosensors, and EEG electrodes, e.g., Ranjbar Koleibi et al., where a compact, ultra-low-power CMOS amplifier with high input impedance was developed for large-scale neural signal recording in implantable systems), biomedical engineering (encompassing biomechanics, wireless telemetry, neurophotonics, and lab-on-chip devices, e.g., Bouffandeau et al., where an impact-based analysis method was validated on soft tissue phantoms, showing superior sensitivity to biomechanical changes compared with existing palpation devices), and machine learning (applied to biomedical signal analysis, e.g., Chellamani et al., where a PPG-based deep learning model achieved highly accurate, non-invasive blood glucose prediction, offering a promising alternative to invasive monitoring).

## 5. An Invitation to Innovate

When assembling this Special Issue, we were struck by the creativity and vision of the authors. Each article offers a glimpse into the future of biosensing, from novel devices and materials to data-driven methodologies, and together they form a mosaic of innovation that transcends traditional boundaries. We encourage researchers, clinicians, and technology developers to engage with the papers, learn from their insights, and build upon their advances. We warmly invite you to explore this collection and join us in pushing the frontiers of (bio)sensing for global health.

## Data Availability

There are no additional raw data for this Editorial.
